# Absolute Reticulocyte Count Acts as a Surrogate for Fetal Hemoglobin in Infants and Children with Sickle Cell Anemia

**DOI:** 10.1371/journal.pone.0136672

**Published:** 2015-09-14

**Authors:** Emily Riehm Meier, Colleen Byrnes, Maxine Weissman, Y. Terry Lee, Jeffery L. Miller

**Affiliations:** 1 Molecular Medicine Branch, National Institute of Diabetes and Digestive and Kidney Diseases, National Institutes of Health, Bethesda, Maryland, United States of America; 2 Center for Cancer and Blood Disorders, Children’s National Medical Center, Washington, D.C., United States of America; 3 Laboratory Medicine Department, Hematology Service, National Institutes of Health, Bethesda, Maryland, United States of America; 4 Department of Pediatrics, The George Washington University School of Medicine and Health Sciences, Washington, D.C., United States of America; Southern Illinois University School of Medicine, UNITED STATES

## Abstract

Hemoglobin switching is largely complete in humans by six months of age. Among infants with sickle cell anemia (HbSS, SCA), reticulocytosis begins early in life as fetal hemoglobin (HbF) is replaced by sickle hemoglobin (HbS). The objective of this study was to determine if absolute reticulocyte count (ARC) is related to HbF levels in a cohort of pediatric SCA patients. A convenience sample of 106 children with SCA between the ages of 1 month and 20 years who were not receiving hydroxyurea or monthly blood transfusions were enrolled in this observational study. Hematologic data, including ARC and HbF levels, were measured at steady state. F-cells were enumerated by flow cytometry. Initial studies compared infants with ARC greater than or equal to 200 K/μL (ARC ≥ 200) based upon the previously reported utility of this threshold as a predictive marker for SCA severity. Mean HbF and F-cell levels were significantly lower in the ARC ≥ 200 group when compared to the ARC < 200 group. Both HbF and F-cell percentages were negatively correlated to ARC in infants and in children between the ages of 1 and 9 years. However, the inverse relationship was lost after the age of 10 years. Overall, decreased expression and distribution of HbF during childhood SCA is well-correlated with increased reticulocyte production and release into the peripheral blood. As such, these data further support the clinical use of reticulocyte enumeration as a disease severity biomarker for childhood sickle cell anemia.

## Introduction

Sickle cell anemia (HbSS, SCA) results from a single amino acid substitution in the beta globin protein. During infancy, fetal hemoglobin (HbF) production is reduced with balanced increases in production of sickle hemoglobin (HbS). HbS polymerizes and causes hemolysis and a shorter life span of circulating red blood cells. The hemolysis further causes decreased oxygen delivery to the tissues [1]. In response, erythropoietin increases erythroblast generation and the release of reticulocytes into the peripheral blood. The cycle of hemolysis causing stressed erythropoiesis/reticulocytosis with additional hemolysis occurs during the lifetime of the host [2]. In rare patients who manifest persistent expression of fetal hemoglobin, less severe SCA phenotypes develop [1].

The protective effects of HbF upon SCA severity continue to motivate basic and clinical research aimed toward therapeutic manipulation of HbF [3]. To date, clinical trials of hydroxyurea, sodium butyrate, and 5-azacytidine have been explored as a pharmaceutical means for HbF-augmentation [4,5]. HbF levels also increase during times of acute stressed hematopoiesis and erythropoiesis, such as during recovery from hematopoietic stem cell transplant [6] or following an acute anemic episode after phlebotomy or transient erythroblastopenia of childhood [7]. Erythroid stress related HbF expression may be due to a change in erythropoietic kinetics [8], signal transduction [9], or other changes in the erythroid environment.

In response to erythropoietin, the absolute number of reticulocytes increases in the peripheral blood [10]. Efforts to induce HbF and thereby improve the SCA phenotype with pulsed doses of erythropoietin resulted in more HbF-containing reticulocytes in adults [11]. In contrast, increased levels of reticulocytosis among infants with SCA are associated with more severe clinical phenotypes during childhood [12,13]. However, the potential relationships between the magnitude of reticulocytosis and HbF expression remain largely unresolved in children with SCA. To further explore this topic, steady-state reticulocyte levels were quantitated in infants and children with SCA and correlated with the expression and distribution of HbF in their blood.

## Materials and Methods

### Subject Eligibility

Discarded samples collected for clinical purposes during steady state (at least 60 days after pRBC transfusion and 30 days after an acute illness that required a hospital visit) were analyzed from a convenience sample of SCA patients receiving supportive care alone at the Children’s National Medical Center Sickle Cell Program. Patients who were receiving hydroxyurea or chronic transfusion therapy were excluded from this study. Consent and assent was obtained prior to enrollment in this observational study, which was approved by the Children’s National Institutional Review Board.

### Hematological and HPLC analyses

Reticulocytes were enumerated using a Sysmex XE 2100 hematology analyzer (Sysmex America, Mundelein, IL). HbF levels were quantitated by high performance liquid chromatography (HPLC) using the VARIANT beta thalassemia short program from Bio Rad Laboratories (Bio Rad, Munich, Germany). HbF level was corrected for the presence of HbA as previously described [14].

### Flow Cytometry

For F-cell enumeration, samples were washed with 0.1% bovine serum albumin in phosphate buffered saline and fixed with 0.05% glutaraldehyde. Cells were washed again and permeablized with 1% triton-X (Caltag Laboratories, Burlingame CA), then immunostained using HbF (HbF-PE; Caltag Laboratories, Burlingame CA) fluorescent antibodies. At least 10,000 gated erythrocytes were analyzed using a FACSAria flow cytometer (Becton Dickinson, San Jose, CA). F-cell percentages were calculated as previously described [14].

### Statistical Analysis

Data analyses were performed using Microsoft Excel 2010 and Social Science Statistical online calculator (www.socscistatistics.com). All statistical tests were two-sided and significance level was set at a probability level of <0.05. Correlation coefficients between ARC and HbF or F-cells were calculated. Student t-test was used to compare means between the two ARC groups.

## Results

### Study Population and Hematologic Data

One hundred six infants and children were enrolled in this observational study. All enrollees had a confirmed diagnosis of HbSS. Average age at the time of analysis was 4.6 years (Table 1, range 0.1–20.0 years). Hemoglobin and absolute reticulocyte count (ARC) had a wide range, likely because of the significant age range of the patient population. Patients were divided into age groups (< 1 year, 1–9 years and 10–20 years). As expected, younger participants had less hemolysis (as evidenced by higher mean hemoglobin and lower mean ARC) than the older children who were studied. The younger patients also had significantly higher HbF and F-cell levels than the two older groups (Table 1). HbF levels were corrected for transfusion in six of the 106 (5.7%) patients, and the differences between uncorrected and corrected HbF values ranged from 0.1–1.5%.

**Table 1:** Demographic and Hematologic Characteristics.

Group	< 1 year	1–9 years	10–20 years	p-value
**n (% of total)**	42 (39.6)	46 (43.4)	18 (17.0%)	
**Age (years)**	0.4±0.2	4.7±2.9	14.0±2.9	0.000
**Female (%)**	21 (50.0)	29 (63.0)	4 (22.2)	
**HbF (%)**	44.5±19.2	18.2±9.3	7.9±4.6	0.000
**Hemoglobin (g/dL)**	8.9±1.7	8.5±1.4	7.8±1.2	0.036
**ARC (K/uL)**	177±119	310±131	352±106	0.000
**F-cells (%)**	91.6±11.2	62.0±24.1	34.4±14.3	0.000

ARC: absolute reticulocyte count, HbF: fetal hemoglobin. Mean values ± standard deviation are listed.

### Reticulocyte Levels are Inversely Associated to HbF Levels in Infants and Children with SCA

**Fig 1:**
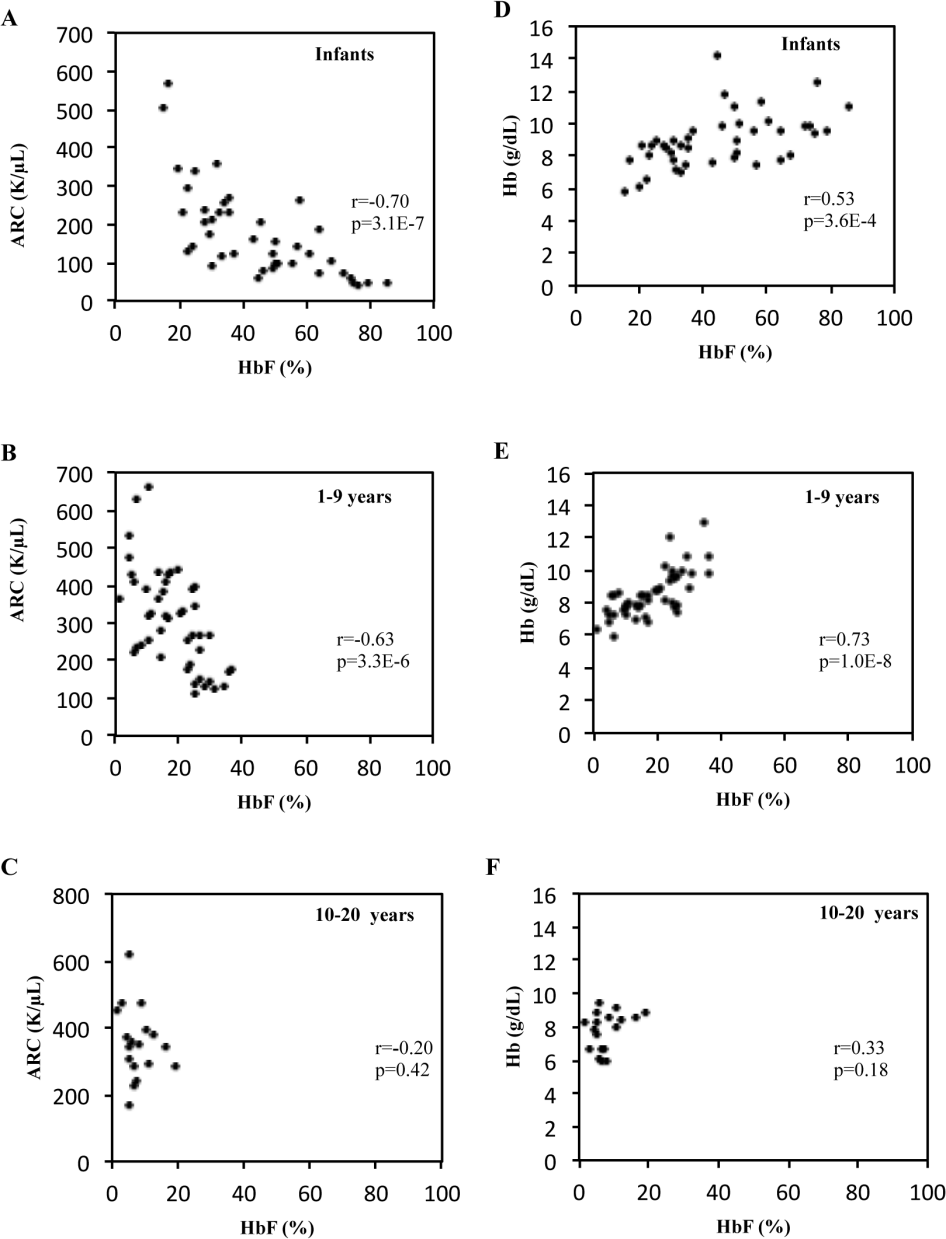
Relationship of ARC or Hemoglobin with HbF According to Age Group. A-C: Correlation of HbF and ARC in patients with SCA who are less than 1 year of age, 1–9 years of age and 10–20 years old, respectively. D-F: Correlation of HbF and hemoglobin in the same age groups, respectively. HbF% is on the x-axis in each panel. Correlation coefficients (r) and p- values are shown in each panel. ARC, Absolute Reticulocyte Count; Hb, hemoglobin. Each dot represents a steady state value for a separate patient.

Studies were performed to determine if the level of reticulocytosis was related to HbF levels (Fig 1A and 1B). HbF was negatively correlated with ARC in infants less than 1 year of age and children between 1 and 9 years of age (r = -0.70, p < 0.0001 and r = -0.63, p < 0.0001, respectively). However, this relationship was lost in older children and adolescents between the ages of 10 and 20 years of age (Fig 1C, r = -0.20, p = 0.42). As expected, hemoglobin levels increased with higher HbF levels in infants and children (r = 0.53, p < 0.0001 and r = 0.73, p < 0.0001, respectively; Fig 1D and 1E). Again, this correlation was lost between the ages of 10 and 20 years (r = 0.33, p = 0.18, Fig 1F).

### F-cells and Reticulocyte Count

**Fig 2:**
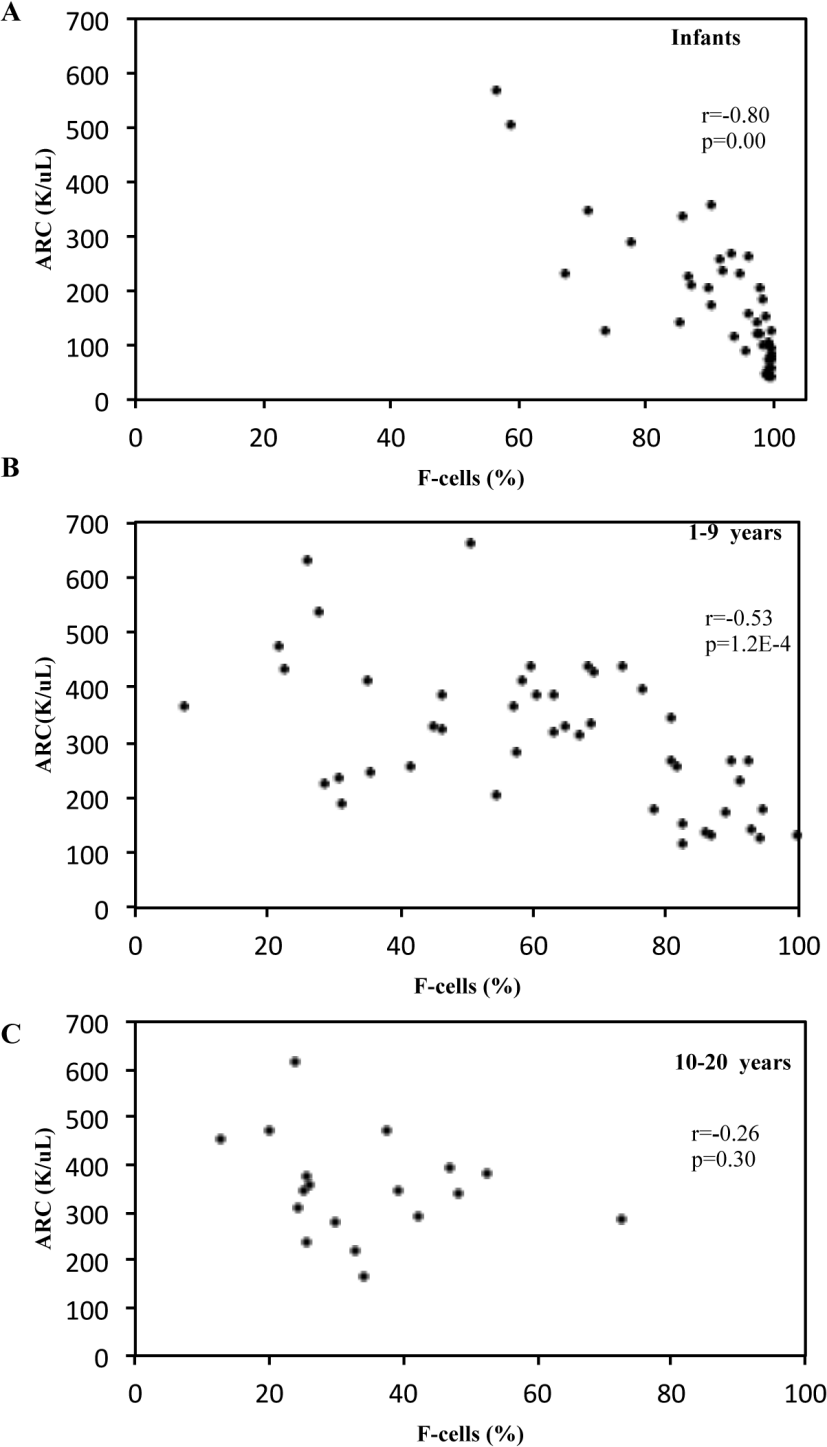
Relationship of ARC and F-cells by Age Group. A. Correlation of F-cell % and ARC in infants with SCA (less than 1 year of age) at steady state. B. Same correlation with children with SCA who are between 1 and 9 years of age and C. between 10 and 20 years of age. ARC, Absolute Reticulocyte Count. Each dot represents a steady state value for a separate patient.

**Fig 3:**
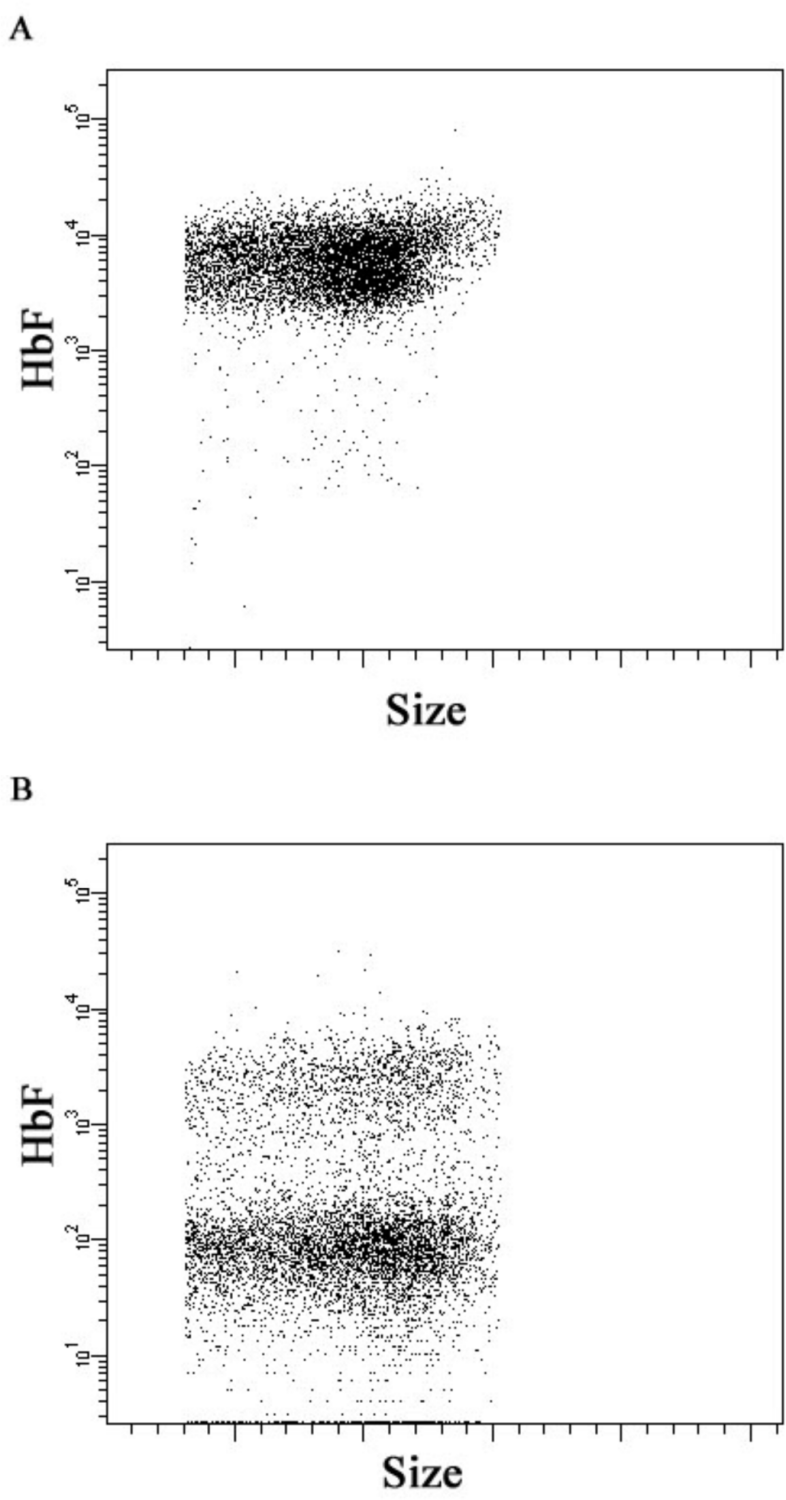
Representative Flow Dot Plots for Pancellular vs. Heterocellular HbF Distribution. A. Erythrocytes from a 3 month old infant with SCA stained with fluorescent antibodies against HbF, revealing a pancellular HbF distribution. B. Representative F-cell staining from an older child with SCA, representative of heterocellular HbF distribution.

A similar negative correlation exists between ARC and F-cells (r = -0.8, p < 0.0001, Fig 2A) in infants with SCA as well as children between the ages of 1 and 9 years (r = -0.53, p < 0.0001, Fig 2B), but was lost in older children and adolescents (r = -0.26, p = 0.30, Fig 2C). Normal ARC (for this report defined as less than 100 K/μL) was limited to a small group of infants with high HbF in a pancellular distribution. As the HbF level dropped below 30%, ARC levels increased to greater than 200 K/μL. Representative flow dot plots of F-cell staining are shown in Fig 3. Infants had nearly pancellular distribution of HbF and F-cells (Fig 3A). Children and adolescents had HbF manifested with a heterocellular pattern of distribution (Fig 3B).

### HbF and F-cell levels in Infants and Children with ARC Levels below and above 200 K/μL

**Fig 4:**
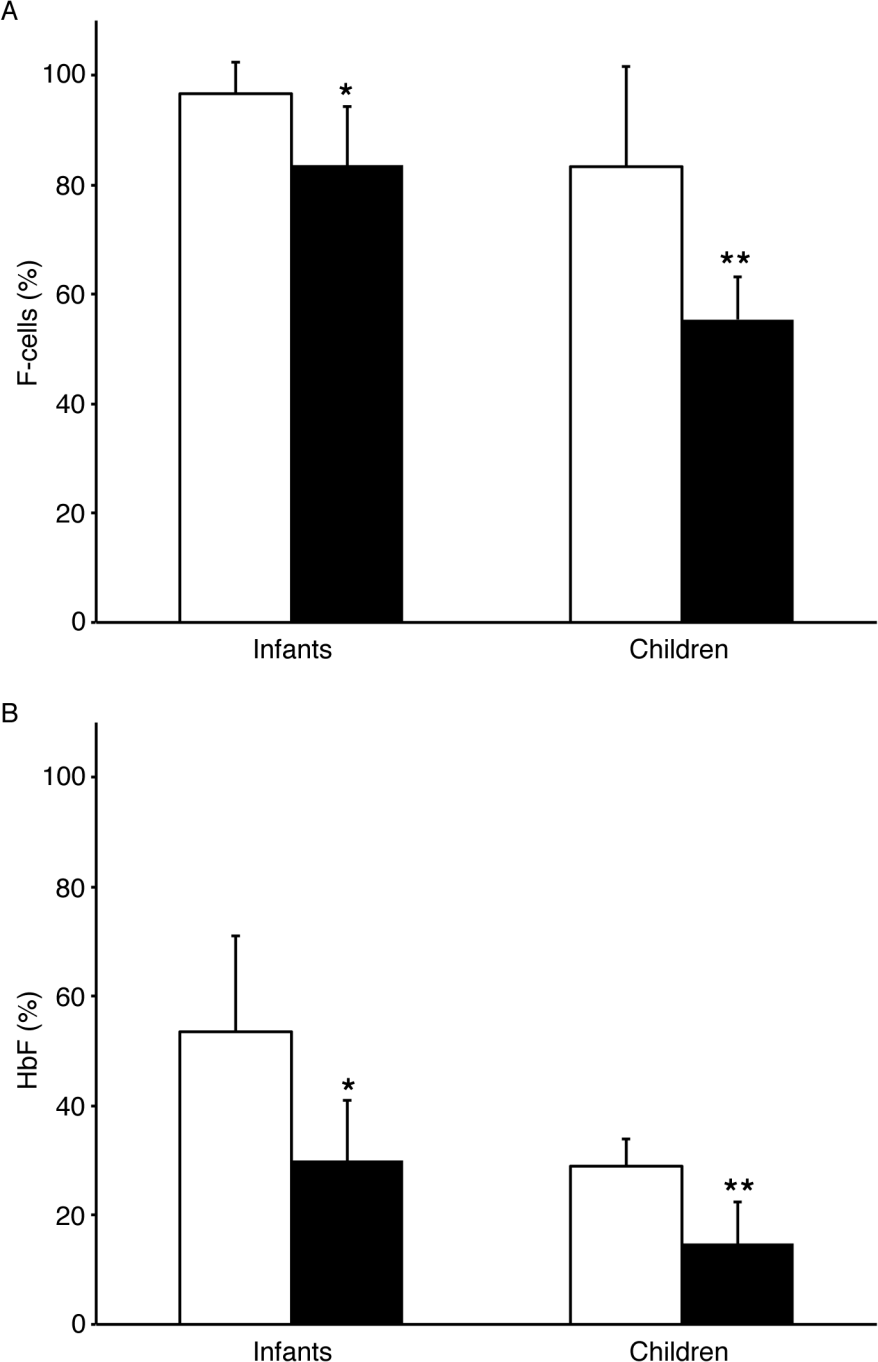
Comparison of F-cell and HbF levels in Infants and Children with ARC<200 K/μL and ARC ≥ 200K/μL. A. F-cell level comparisons in infants less than 1 year of age and children between ages 1–9 years with an ARC < 200 K/μL (open bars) and ARC ≥ 200 K/μL (shaded bars) (*p = 6.2 E-5, **p = 1.7E-6). B. Comparison of HbF levels in infants and children ages 1–9 years and ARC < 200 K/μL (open bars) and ARC ≥ 200 K/μL (shaded bars) (*p = 2.2E-5,**p = 7.4E-7). Standard deviation bars are included.

Based on prior studies [12,13], we also grouped infants and children according to their ARC (less than 200 K/μL and greater than or equal to 200 K/μL). Only one patient in the 10–20 year age group had an ARC less than 200 K/μL. The group of infants who had an ARC less than 200K/μL had significantly higher HbF levels (53.5 ± 17.6% vs. 29.96 ± 10.9%, p = 2.2 E-5) as well as broader distribution of F-cells (96.6 ± 5.7% vs. 83.5 ± 13.2%, p = 6.2 E-5) than infants with ARC greater than or equal to 200K/μL (Fig 4A). Differences in HbF and F-cell levels between children (ages 1 and 9 years) that were grouped according to ARC were even more striking [HbF: 29.0 ± 4.9% vs. 14.8 ± 7.7%, p = 7.4E-7; F-cells: 83.3 ± 18.4% vs. 55.4 ± 21.9%, p = 1.7E-6 for ARC < 200K/μL and ARC ≥ 200K/μL groups respectively (Fig 4B)].

## Discussion

The concentration and distribution of HbF within individual erythrocytes is an important determinant for disease severity in SCA patients [15,16]. According to this study, increased ARC is associated with pronounced decreases in HbF and F-cell levels among infants with SCA. Less robust correlations between HbF levels and ARC are maintained through age 10 years, but are lost in adolescence and young adulthood. For comparison, S1 Fig contains age correlation data regarding HbF, ARC, F-cell, and hemoglobin for the entire cohort. Our data confirm that in early infancy, HbF is present in a pancellular distribution in nearly all erythrocytes. Consistent with other reports, the distribution of HbF-containing cells decreases as HbF is silenced in these children [17, 18]. Our data suggest that decreases in bulk HbF and the transition to heterocellular HbF distribution correlate well with increasing erythroid stress as measured by ARC. Our data also highlight the gradual change in HbF and F-cell levels throughout infancy and childhood in patients with SCA. HbF and F-cell levels in the adolescents included in our study are similar to adult levels [19]. We previously reported that increased reticulocytosis during early infancy is associated with early hospitalizations, stroke and death [12,13]. Given the negative correlations between HbF, F-cell distribution and ARC in infants and children in this study, ARC may serve as a surrogate for HbF levels and distribution in patients with SCA up to 9 years of age. The potential for implementation of less expensive or more widely available hematological assays may be especially relevant in resource poor countries where the majority of infants with SCA are born [20].

Among infants and children with SCA, stressed erythropoiesis and increased reticulocytosis is associated with lower HbF levels as well as a reduced distribution within circulating erythrocytes. This is most likely due to the ability of HbF to inhibit HbS polymerization, leading to decreased hemolysis and, ultimately, a lower ARC. Conversely, regulation of HbF by steady state erythropoietic stress is not sufficient to overcome the deleterious effects of HbS in this cohort. These data are consistent with the minor effects upon HbF achieved with pulsed erythropoietin, hypoxia, or phlebotomy in humans [7,8].

Our prior studies demonstrated a predictive value of decreased reticulocytosis for decreased disease severity in infants with SCA [12,13]. The level of reticulocytosis produced during the first six months of life may serve as a very early marker of disease severity among infants with SCA, and the reticulocyte adhesion characteristics may contribute to the early pathophysiology as well. Here, high HbF levels and low ARC (< 200K/μL) are rarely found in children over the age of 10 years. An important limitation of this retrospective study is the small group of older children and adolescents included. Those patients who were receiving hydroxyurea or chronic blood transfusions were not included in this study due to potentially confounding effects of those therapies upon ARC levels. As such, the older children and adolescents included in our study may have less severe disease, or they may not be receiving hydroxyurea due to a family preference [1]. We therefore hypothesize that the predictive value of reticulocytosis for disease severity in SCA may be lost during childhood for those patients treated with supportive care, but larger prospective studies are needed to further study the role of reticulocytosis in risk stratification for SCA patients of all ages. Consistent with models comparing acute and chronic erythroid stress [8], the data also imply that mechanisms that activate HbF ex vivo or in the setting of bone marrow ablation may be distinct from the steady-state of SCA.

While two thirds of infants had ARC levels below 200K/μL, only 1 of the 18 (~5%) SCA patients between 10 and 20 years old had a similar low level of reticulocytosis. As evidenced here, under conditions of chronic erythroid stress, there appears to be an ongoing loss and silencing of HbF production that lasts well past infancy [21]. As fetal cells with high HbF levels are replaced with HbS-containing erythrocytes with low levels of HbF, anemia ensues from the resultant sickling and hemolysis. Thus begins a vicious cycle of anemia, tissue hypoxia, and reticulocytosis with continued production of HbS-containing cells. Based upon the more rapid destruction of these newly produced HbS cells, higher bulk levels of HbF may be expected in the peripheral blood of newborns during this transition period until the switching phenomenon is complete. An inverse relationship develops during childhood between the level of reticulocytosis (ARC) with HbF and F-cell distribution.

Overall, infants and children less than 10 years of age with SCA and increased ARC have significantly lower HbF and F-cell levels compared to age-matched SCA patients with less pronounced reticulocytosis. Among this cohort, the negative correlation between ARC and HbF and F-cells was age-dependent and lost during childhood.

## Supporting Information

S1 Fig Non-age stratified hematologic data for the entire cohort. Correlation of A. HbF (%), B. Hb (g/dL), C. F-cells (%), D. ARC (K/μL) with age in years. HbF, Fetal hemoglobin; Hb, hemoglobin; ARC, Absolute Reticulocyte Count. Each dot represents a steady state value for a separate patient.
(TIF)
