# Supplementary figures and images for: Absolute Reticulocyte Count Acts as a Surrogate for Fetal Hemoglobin in Infants and Children with Sickle Cell Anemia
Source: PLoS One. 2015 Sep 14;10(9):e0136672. doi: 10.1371/journal.pone.0136672 (PMC4569336; doi:10.1371/journal.pone.0136672)

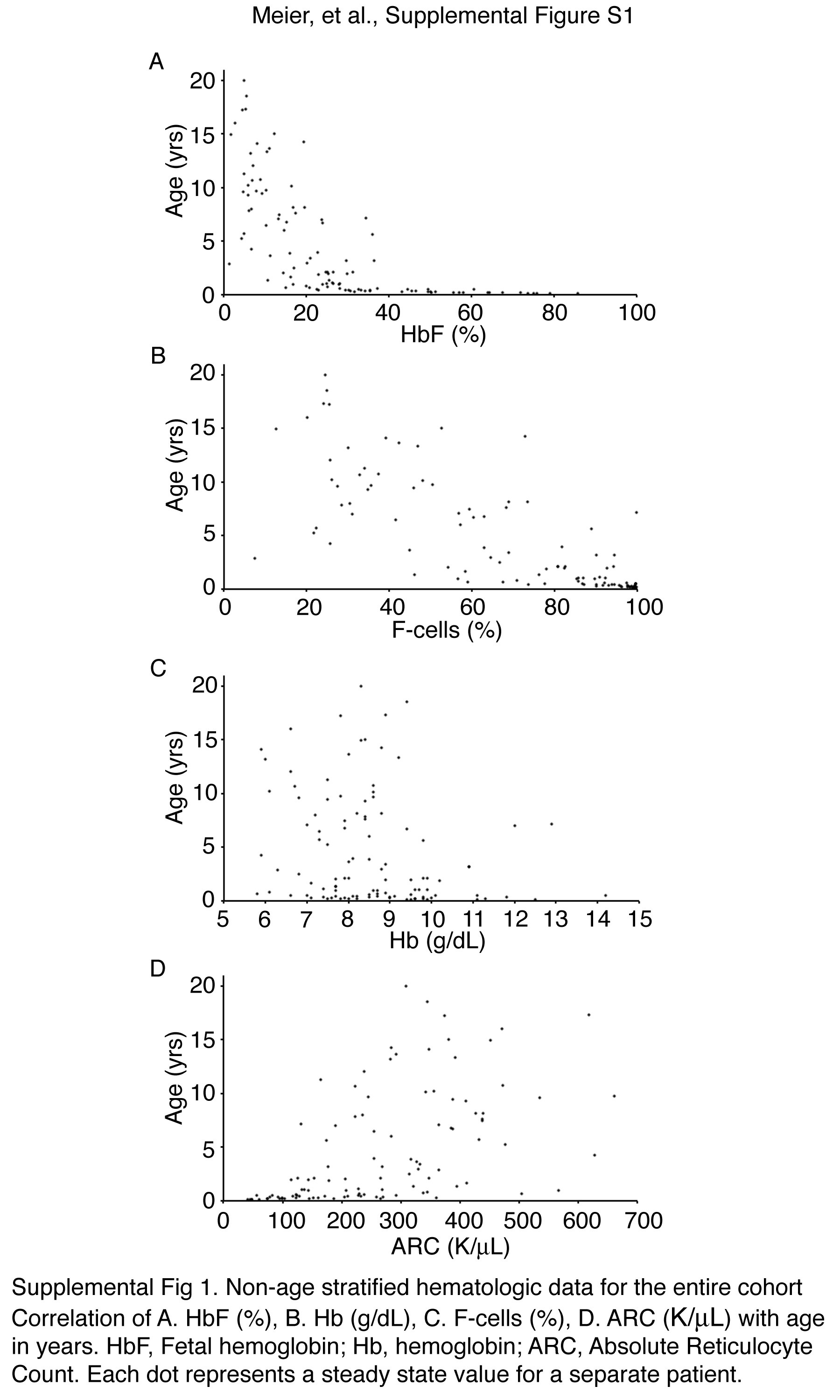

Supplement: S1 Fig — Correlation of A. HbF (%), B. Hb (g/dL), C. F-cells (%), D. ARC (K/μL) with age in years. HbF, Fetal hemoglobin; Hb, hemoglobin; ARC, Absolute Reticulocyte Count. Each dot represents a steady state value for a separate patient. (TIF) [file pone.0136672.s001.tif]
